# Gum Arabic Ameliorates Impaired Coagulation and Cardiotoxicity Induced by Water-Pipe Smoke Exposure in Mice

**DOI:** 10.3389/fphys.2019.00053

**Published:** 2019-02-25

**Authors:** Abderrahim Nemmar, Suhail Al-Salam, Sumaya Beegam, Priya Yuvaraju, Badreldin H. Ali

**Affiliations:** ^1^Department of Physiology, College of Medicine and Health Sciences, United Arab Emirates University, Al Ain, United Arab Emirates; ^2^Department of Pathology, College of Medicine and Health Sciences, United Arab Emirates University, Al Ain, United Arab Emirates; ^3^Department of Pharmacology and Clinical Pharmacy, College of Medicine and Health Sciences, Sultan Qaboos University, Muscat, Oman

**Keywords:** water-pipe smoke, nose-only exposure, Gum Arabic, heart, oxidative stress, inflammation, thrombosis

## Abstract

Water-pipe smoking (WPS) is prevalent in the East and elsewhere. WPS exposure is known to induce thrombosis and cardiovascular toxicity involving inflammation and oxidative stress. Here, we have investigated the effect of Gum Arabic (GA), a prebiotic with anti-oxidant, anti-inflammatory and cytoprotective properties, on WPS exposure (30 min/day for 1 month) on coagulation and cardiac homeostasis, and their possible underlying mechanisms in mice. Animals received either GA in drinking water (15%, *w*/*v*) or water only for the entire duration of study. GA significantly mitigated thrombosis in pial microvessels *in vivo*, platelet aggregation *in vitro*, and the shortening of prothrombin time induced by WPS exposure. The increase in plasma concentrations of fibrinogen, plasminogen activator inhibitor-1 and markers of lipid peroxidation, 8-isoprostane and malondialdehyde, induced by WPS were significantly reduced by GA administration. Moreover, WPS exposure induced a significant increase in systolic blood pressure and the concentrations of the pro-inflammatory cytokines tumor necrosis factor-α and interleukin 1β in heart homogenates. GA significantly alleviated these effects, and prevented the decrease of reduced glutathione, catalase and total nitric oxide levels in heart homogenates. Immunohistochemical analysis of the hearts showed that WPS exposure increased nuclear factor erythroid-derived 2-like 2 (Nrf2) expressions by cardiac myocytes and endothelial cells, and these effects were potentiated by the combination of GA and WPS. WPS also increased DNA damage and cleaved caspase 3, and GA administration prevented these effects. Our data, obtained in experimental murine model of WPS exposure, show that GA ameliorates WPS-induced coagulation and cardiovascular inflammation, oxidative stress, DNA damage and apoptosis, through a mechanism involving Nrf2 activation.

## Introduction

Water-pipe smoking (WPS) is prevalent in Eastern countries and is nowadays increasing globally, including in Europe and North America, and is becoming a growing public health issue (Maziak et al., 2017). WPS is the most popular method of tobacco use among young people and women in Middle-Eastern countries (Maziak et al., 2017; Al Ghobain et al., 2018). In the United Kingdom, it has been reported that 1% of adults are regular water-pipe tobacco smokers (Grant et al., 2014). In the United States, data from a national survey estimated that 7.8% of young adults aged 18–24 years are current water-pipe tobacco smokers (Salloum et al., 2015). WPS patterns in the United States have been reported to be comparable across genders, with the exception that women have been more regularly reported smoking water-pipe in cafés and being selectively water-pipe smokers (Maziak et al., 2015). WPS is considered as a gateway for cigarette smoking (CS). In fact, it has been reported that there is a high probability for occasional or frequent WPS to turn into regular cigarette smokers (Jensen et al., 2010). Although reports have stated that WPS may possibly lead to dependence, it is, however, perceived as less toxic and addictive than CS (Maziak et al., 2017). Adverse health effects related WPS include respiratory and cardiovascular diseases, and cancer (Maziak et al., 2017).

The impact of CS on the cardiovascular system has been largely described. Nevertheless, reports on the adverse cardiovascular effects of WPS exposure remain relatively limited. It has been shown that WPS exposure augments respiratory rate, heart rate, mean systolic and diastolic blood pressure, and CO levels (Kadhum et al., 2014). Moreover, it has been shown that chronic exposure to WPS in humans causes a significant increase in the risk of coronary artery disease (Sibai et al., 2014). We have recently reported that both short-term and long-term exposure to WPS causes cardiovascular oxidative stress, inflammation, and thrombotic disorders in mice (Nemmar et al., 2013b, 2015a, 2017b).

Epidemiological and clinical studies revealed that an increased intake of dietary natural phytochemicals is consistently linked with protection from chronic cardiovascular and neurodegenerative diseases (Hooper et al., 2008; Islam et al., 2016). Gum Arabic (also known as gum Acacia, GA) is the edible and dried gummy exudates from the *Acacia senegal* tree (Ali et al., 2009). It is a well-known safe dietary fiber with various applications in the food, pharmaceutical and cosmetic industries (Ali et al., 2009). In mice and rats, GA has been reported to act as an anti-oxidant, anti-inflammatory and cytoprotective agent, protecting against hepatic and renal toxicities (Gamal el-din et al., 2003; Ali et al., 2013b, 2014; Al Za’abi et al., 2018). Moreover, it has been shown that GA has beneficial effects in patients with chronic kidney diseases, sickle cell anemia, type 2 diabetes and obesity (Babiker et al., 2012; Elamin et al., 2017; Kaddam et al., 2017).

As we have established that the cardiovascular effects of WPS exposure in mice involve oxidative stress and inflammation (Nemmar et al., 2013b, 2015b, 2017b), we have, consequently, postulated that intake of GA would lead to a reduction in the cardiovascular injurious effects of WPS exposure. Therefore, the present study was designed to evaluate the possible ameliorative effects of GA consumption on the cardiovascular toxicity induced by WPS, and the mechanisms underlying these effects. This is the first study on such interaction.

## Materials and Methods

### Animals and Treatments

All studies involving animals were conducted in accordance with and after approval of the animal research ethics committee of the United Arab Emirates University (ERA_2017_5625). A total number of 91 mice were used to assess various biochemical, histological, and physiological parameters.

### WPS Exposure

C57BL/6 mice (Taconic Farms Inc., Germantown, NY, United States) were housed in a conventional animal house and maintained on a 12-hour light-dark cycle (lights on at 6:00 am). The animals were placed in cages and supplied with pelleted food and water *ad libitum*. Following 1 week of acclimatization, animals were randomly divided into 4 groups, air (control), WPS, GA+air, and GA+WPS.

Mice were placed in soft restraints and connected to the exposure tower (Ali et al., 2017; Nemmar et al., 2017b). The animals were exposed to either WPS or air through their noses using a nose-only exposure system connected to a water-pipe (InExpose System, SCIREQ, Canada). Animals were exposed to a commercially available apple-flavored tobacco (Al Fakher Tobacco Trading, Ajman, UAE). Tobacco was lit with an instant light charcoal disk (Star, 3.5 cm diameter and 1 cm width). As is the case for human use, the smoke from the water-pipe passes first through the water before it is drawn into the exposure tower. The exposure regimen is controlled by a computerized system (InExpose System, SCIREQ, Canada). A computer-controlled puff was generated every minute, leading to a 2 s puff duration of WPS exposure followed by 58 s of fresh air. The duration of an exposure session was 30 min/day (Nemmar et al., 2013b, 2016a). This was selected from a published work that has assessed the cardiorespiratory effects of WPS in healthy subjects (Hakim et al., 2011). Mice were exposed for 1 month either to air or WPS without or with GA (Sigma, St Louis, MO, United States) added to the drinking water at a concentration of 15% (*w*/*v*) (Ali et al., 2013b).

### Experimental Pial Cerebral Arterioles Thrombosis Model

*In vivo* pial arteriolar and venular thrombogenesis was assessed at the end of the 1 month exposure period to either WPS or air with or without GA treatment, according to a previously described technique (Nemmar et al., 2009, 2013a). Briefly, the trachea was intubated after induction of anesthesia with urethane (1 mg/g, i.p.), and a 2F venous catheter (Portex, Hythe, United Kingdom) was inserted in the right jugular vein for the administration of fluorescein (Sigma, St. Louis, MO, United States). Thereafter, a craniotomy was first performed on the left side, using a microdrill, and the dura was stripped open. Only untraumatized preparations were used, and those showing trauma to either microvessels or underlying brain tissue were discarded. The animals were then placed on the stage of a fluorescence microscope (Olympus, Melville, NY, United States) attached to a camera and DVD recorder. A heating mat was placed under the mice and body temperature was raised to 37°C, as monitored by a rectal thermoprobe connected to a temperature reader (Physitemp Instruments, NJ, United States). The cranial preparation was moistened continuously with artificial cerebrospinal fluid of the following composition (mM): NaCl = 124, KCl = 5, NaH_2_PO_4_ = 3, CaCl = 2.5, MgSO_4_ = 2.4, NaHCO_3_ = 23 and glucose = 10, pH 7.3–7.4 (El Sabban and Radwan, 1997). A field containing arterioles and venules 15–20 μm in diameter was chosen. Such a field was taped prior to and during the photochemical insult. The photochemical insult was carried out by injecting fluorescein (200 mg/kg) via the jugular vein, which was allowed to circulate for 30–40 s. The cranial preparation was then exposed to stabilized mercury light. The combination produces endothelium injury of the arterioles and venules. This, in turn, causes platelets to adhere at the site of endothelial damage and then aggregate. Platelet aggregates and thrombus formation grow in size until complete arteriolar or venular occlusion. The time from the photochemical injury until full vascular occlusion (time to flow stop) in arterioles and venules was measured in seconds. At the end of the experiments, the animals were euthanized by an overdose of urethane.

### Systolic Blood Pressure (SBP) Measurement

Systolic Blood Pressure was measured using a computerized non-invasive tail-cuff manometry system (ADInstrument, Colorado Springs, CO, United States) (Nemmar et al., 2013b). To avoid procedure-induced anxiety, mice were trained for 3 consecutive days before the experimental procedure.

### Blood Collection

At the end of the exposure period, animals were anesthetized intraperitoneally with sodium pentobarbital (45 mg/kg), and then blood was drawn from the inferior vena cava in EDTA (4%). The blood was centrifuged for 15 min at 4°C at 900 *g*, and the plasma samples obtained were stored at –80°C pending analysis.

### Platelet Aggregation in Mouse Whole Blood

In mice exposed to either WPS or air, with or without GA treatment, the platelet aggregation assay in whole blood was performed with slight modification, as described before (Nemmar et al., 2008, 2014). After anesthesia, blood was withdrawn from the inferior vena cava and placed in citrate (3.2%), and 100-μl aliquots were added to the well of a coagulometer (MC 1 VET, Merlin, Lemgo, Germany). The blood samples were incubated at 37.2°C with adenosine diphosphate (ADP) (1 μM) for 3 min, and then stirred for another 3 min. At the end of this period, 25-μl samples were removed and fixed on ice in 225 ml cellFix (Becton Dickinson, Franklin Lakes, NJ, United States). After fixation, single platelets were counted in a VET ABX Micros with mouse card (ABX, Montpellier, France). The degree of platelet aggregation, assessed as a fall in single platelets counted in the presence of ADP, was determined in whole blood collected from mice exposed to either WPS or air with or without GA treatment. These results were compared with those obtained from untreated (without ADP but with saline) whole blood obtained from control (unexposed) mice.

### Prothrombin Time (PT) Measurement in Plasma *in vitro*

At the end of exposure to either WPS or air with or without GA treatment, mice were anesthetized, blood was withdrawn from the inferior vena cava and placed in citrate solution (3.2%) (ratio of blood to anticoagulant: 9:1). The PT was measured (Nemmar et al., 2013b) on freshly collected platelet-poor plasma with human relipidated recombinant thromboplastin (Recombiplastin; Instrumentation Laboratory, Orangeburg, NY, United States) in combination with a coagulometer (MC 1 VET, Merlin, Lemgo, Germany).

### Measurement of Fibrinogen and Plasminogen Activator Inhibitor-1 Concentrations in Plasma

The plasma concentrations of fibrinogen and PAI-1 were measured using ELISA kits (Molecular Innovation, Southfield, MI, United States).

### Measurement of 8-Isoprostane and Malondialdehyde (MDA) Levels in Plasma

The plasma concentrations of 8-isoprostane were measured according to the vendor’s protocol (Cayman Chemicals, MI, United States). NADPH-dependent membrane lipid peroxidation was measured as thiobarbituric acid reactive substance using MDA as standard (Sigma-Aldrich Fine Chemicals, St Louis, MO, United States) (Nemmar et al., 2015a,b).

### Measurement of Tumor Necrosis Factor α (TNFα), Interleukin 1β (IL1β), Catalase, Glutathione (GSH) and Total Nitric Oxide (NO) Levels in Heart Homogenates

Heart homogenates for the measurement of markers of inflammation and oxidative stress were prepared as described before (Nemmar et al., 2013a). The concentrations of TNFα and IL1β were determined using commercially available kits (Duo Set, R&D systems, Minneapolis, MN, United States). The activity of catalase (Cayman Chemicals, MI, United States) and GSH concentration (Sigma-Aldrich Fine Chemicals, St Louis, MO, United States) were measured according to the vendors’s protocols. The determination of nitric oxide (NO) was performed with a total NO assay kit from R&D systems (Minneapolis, MN, United States), which measures the more stable NO metabolites NO_2_^-^ and NO_3_^-^ (Wennmalm et al., 1993; Tsikas, 2005).

### Histopathology and Immunohistochemistry

Hearts were collected following sacrifice, washed with ice-cold saline, blotted with filter paper, weighed and fixed in 10% neutral formalin. Each heart was coronally sectioned into 4 pieces, casseted and dehydrated in increasing concentrations of ethanol, cleared with xylene and embedded in paraffin. Three-micrometer sections were prepared from paraffin blocks and stained with hematoxylin and eosin. The stained sections were evaluated blindly using light microscopy by a histopathologist who participated in this project (SA).

Regarding immunohistochemistry, five-micrometer sections were cut, de-waxed with xylene and rehydrated with graded alcohol. The slides were then placed in a 0.01 M citrate buffer solution (pH = 6.0) and pre-treatment procedures to unmask the antigens were performed in a water bath for 60 min. Sections were treated with peroxidase and protein block for 15 min each and then incubated with the primary antibodies anti-nuclear factor erythroid-derived 2-like 2 (Nrf2) (rabbit polyclonal antibody, Abcam, United States) for 1 h. After conjugation with primary antibodies, sections were washed and then incubated with Dako REAL^TM^ EnVision^TM^/HRP for 1 h at room temperature (DAKO, Agilent, United States), followed by washing and addition of DAB chromogen (DAKO, Agilent, United States). Sections were then counter stained with hematoxylin. Appropriate positive controls were used. For the negative control, the primary antibody was not added to sections and the whole procedure was carried out in the same manner as mentioned above. The immunohistochemical staining of heart tissue for Nrf2 was scored semi-quantitatively and blindly by our histopathologist on a scale of 0–4 according to the percentage of staining of heart muscles in 4 slides of each specimen, and each slide contained 4 equal coronal slices of the heart. A score of 0 was assigned if the expression was between 0–10%, 1 for 11–25%, 2 for 26–50%, 3 for 51–75% and 4 for more than 75% (Nemmar et al., 2017a, 2018a).

### Assessment of DNA Damage in the Heart by COMET Assay

Immediately after sacrifice, the heart was removed from each animal. Single-cell suspensions of the different hearts were obtained according to a previously described method (de Souza et al., 2014; Nemmar et al., 2016b). Each heart collected was washed in a chilled Roswell Park Memorial Institute (RPMI) 1640 medium containing DMSO = 15% (*v*/*v*) and NaCl = 1.8% (*w*/*v*), placed in 1.5 ml medium and chopped into fine pieces in a Petri dish using scissors. The pieces were allowed to settle and the supernatant was collected in a 15 ml tube. The obtained cell suspension was centrifuged at 900 *g* for 5 min at 4°C. The supernatant was discarded and the pellets were resuspended in 0.5 ml of the medium. The cell suspensions were mixed with low melting point agarose solution (0.65%) and spread onto agarose (1.5%)–precoated microscope slides. For each treatment, 5 slides were prepared, which were incubated in ice cold lysis buffer (2.5 M NaCl, 10 mM Tris, 100 mM EDTA, 1% Triton X-100 and 10% DMSO) at 4°C for at least 1 h to remove the cell membranes. After the incubation, slides were placed in a horizontal electrophoresis unit and incubated in electrophoresis buffer (0.2 M EDTA, 5 M NaCl, pH 10) for 20 min for DNA unwinding and the expression of alkali labile sites. Then, electrophoresis was conducted for 20 min at 25 V and 300 mA. After that, the slides were neutralized with Tris buffer (0.4 M Trizma base, pH 7.5) for 5 min and washed with methanol. Then the slides were stained with propidium iodide, as previously described (Olive et al., 1994; Nemmar et al., 2016a). All these steps were performed in darkness to prevent additional DNA damage. The slides were mounted on a fluorescent microscope and cell scoring was performed. Fifty cells from each treatment were scored and analyzed for DNA migration and the average of the 5 slides from each group was calculated. The measurement of length of the DNA migration (i.e., diameter of the nucleus plus migrated DNA) was calculated using image analysis Axiovision 3.1 software (Carl Zeiss, Canada) (Hartmann and Speit, 1997; Nemmar et al., 2016a).

### Measurement of Cleaved Caspase 3 in Heart Homogenates

Heart homogenates for the measurement of cleaved caspase 3 in mice exposed to either air or WPS with or without GA treatment were prepared as described before (Nemmar et al., 2017b). Cleaved caspase 3 was quantified with mouse (Asp175) DuoSet IC ELISA kit from R&D systems (Minneapolis, MN, United States).

### Statistics

One-way analysis of variance (ANOVA) and all graphs were produced using GraphPad Prism Version 5 for Windows software (GraphPad Software Inc., San Diego, CA, United States). To determine whether parameters were normally distributed, the Kolmogorov–Smirnov statistic normality test was applied. Data were expressed as means ± SEM. To adjust the *P* values obtained from multiple comparisons, we used the Bonferroni–Holm method. This method is based on a sequential approach whose goal is to increase the power of the statistical tests while keeping under control the family-wise Type I error (Holm, 1979; Aickin and Gensler, 1996). The R software version 3.0.3 (R Core Team, 2014) was used for this analysis. The Bonferroni–Holm method was carried out using the p.adjust function from the R package stats. *P* values ≤ 0.05 were considered to be significantly different.

## Results

### Effect of GA on WPS-Induced Thrombotic Events *in vivo*

**FIGURE 1:**
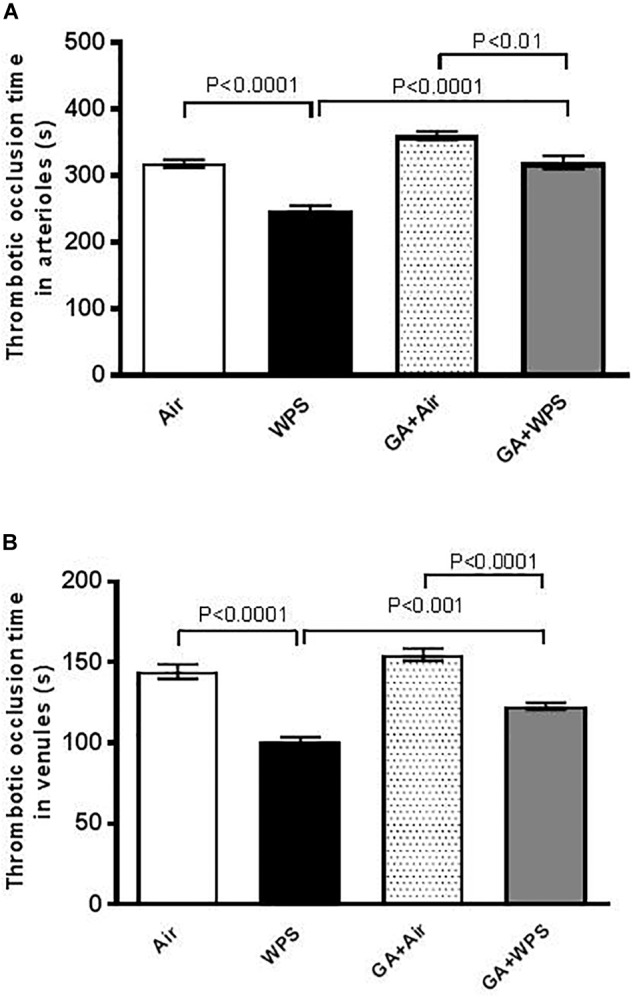
Thrombotic occlusion time in pial arterioles **(A)** and venules **(B)** in mice at the end of 1 month exposure period to either air (*n* = 8) or water-pipe smoke (WPS, *n* = 7) or gum Arabic (GA) administration (15% *w*/*v* in the drinking water) + air (*n* = 8) or GA + WPS (*n* = 7). Data are mean ± SEM.

The thrombotic occlusion time in pial arterioles and venules was significantly shortened by WPS exposure (*P* < 0.0001), compared with the air-exposed group. GA treatment induced a significant mitigation in the prothrombotic events in pial arterioles (*P* < 0.0001) and venules (*P* < 0.001) induced by WPS (Figure 1).

### Effect of GA on WPS-Induced Platelet Aggregation in Whole Blood and Shortening in PT *in vitro*

**FIGURE 2:**
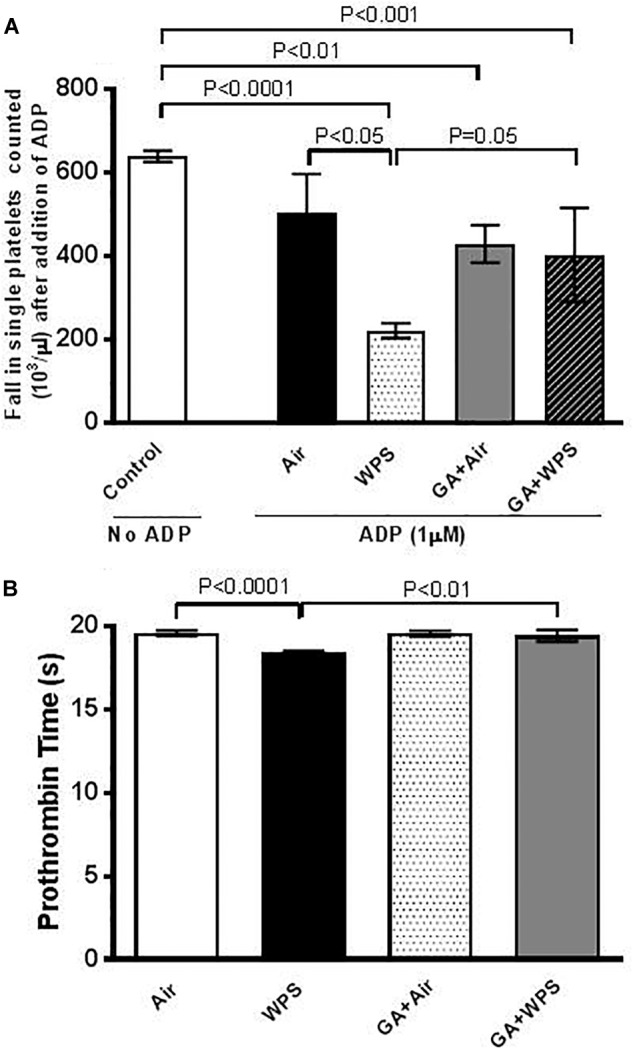
*In vitro* platelet aggregation in whole blood obtained from either control untreated mice (*n* = 5) without the addition of adenosine diphosphate (ADP) or after the addition of ADP (1 μM, **A**) to whole blood collected from mice at the end of 1 month exposure period to either air (*n* = 5) or WPS (*n* = 5) or GA administration (15% *w*/*v* in the drinking water) + air (*n* = 7) or GA + WPS (*n* = 7). Prothrombin time (PT, **B**) at the end of 1 month exposure period to either air (*n* = 4) or WPS (*n* = 4) or GA administration (15% *w*/*v* in the drinking water) + air (*n* = 4) or GA + WPS (*n* = 4). Data are mean ± SEM.

Figure 2A shows that, after incubation with ADP, there was a significant platelet aggregation in whole blood of mice exposed to WPS for 1 month compared with that collected from mice exposed to air (*P* < 0.05). GA treatment prevented the proaggregatory effect of WPS exposure (*P* = 0.05).

Likewise, compared with the air-exposed group, WPS exposure induced a slight but statistically significant shortening in PT (*P* < 0.0001). The latter effect was significantly alleviated by GA administration (*P* < 0.01) (Figure 2B).

### Effect of GA on WPS-Induced Increase in Concentrations of Fibrinogen and PAI-1

**FIGURE 3:**
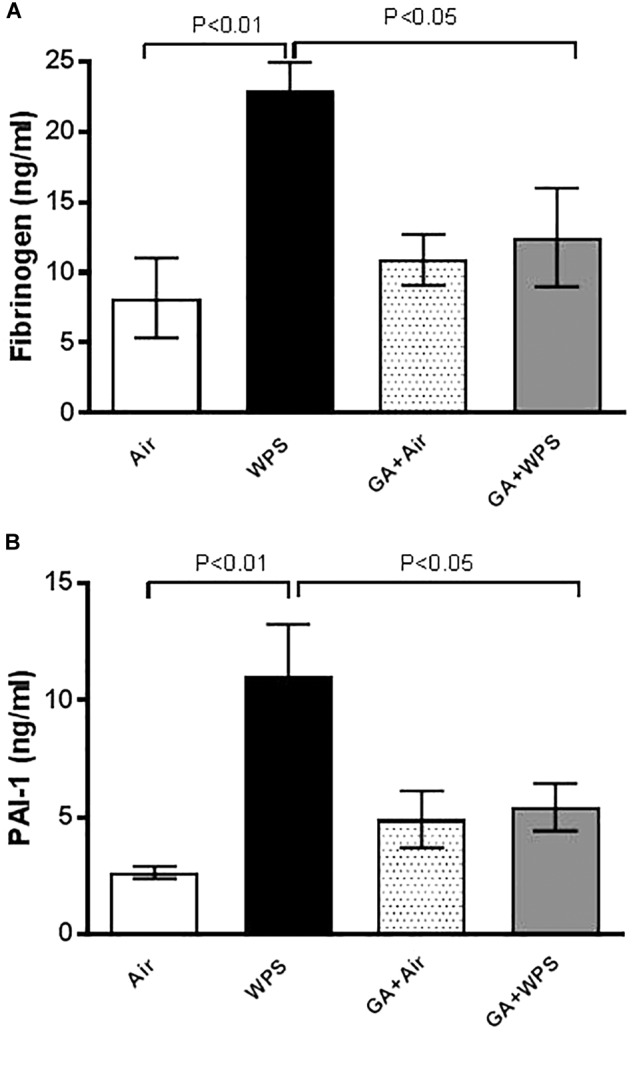
Plasma concentrations of fibrinogen **(A)** at the end of 1 month exposure period to either air (*n* = 8) or WPS (*n* = 7) or GA administration (15% *w*/*v* in the drinking water) + air (*n* = 7) or GA + WPS (*n* = 6). Plasminogen activator inhibitor-1 (PAI-1, **B**) concentrations in the plasma at the end of 1 month exposure period to either air (*n* = 8) or WPS (*n* = 8) or GA administration (15% *w*/*v* in the drinking water) + air (*n* = 7) or GA + WPS (*n* = 7). Data are mean ± SEM.

Figure 3A illustrates that the exposure to WPS caused a significant increase in the concentration of fibrinogen (*P* < 0.01), and that the treatment with GA significantly prevented this effect (*P* < 0.05).

Similarly, compared with the air group, WPS exposure triggered a significant increase in the concentration of PAI-1 (*P* < 0.01). The latter effect was abolished by GA administration (*P* < 0.05) (Figure 3B).

### Effect of GA on WPS-Induced Increase in SBP

**FIGURE 4:**
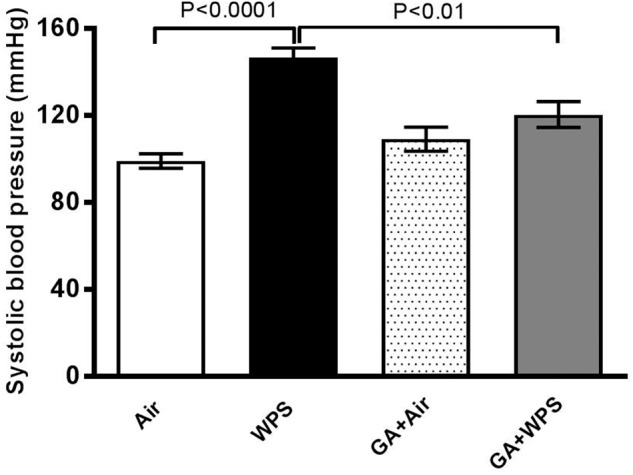
Systolic blood pressure measured in mice at the end of 1 month exposure period to either air (*n* = 8) or WPS (*n* = 7) or GA administration (15% *w*/*v* in the drinking water) + air (*n* = 8) or GA + WPS (*n* = 7). Data are mean ± SEM.

Figure 4 shows that WPS exposure for 1 month caused a significant increase in SBP, compared with the air group (*P* < 0.0001). GA treatment was effective in decreasing the elevation of SBP induced by WPS, as there was a significant reduction in SBP in the GA+WPS group compared with WPS (*P* < 0.01).

### Effect of GA on WPS-Induced Increase in Plasma Concentrations of 8-Isoprostane and MDA

**FIGURE 5:**
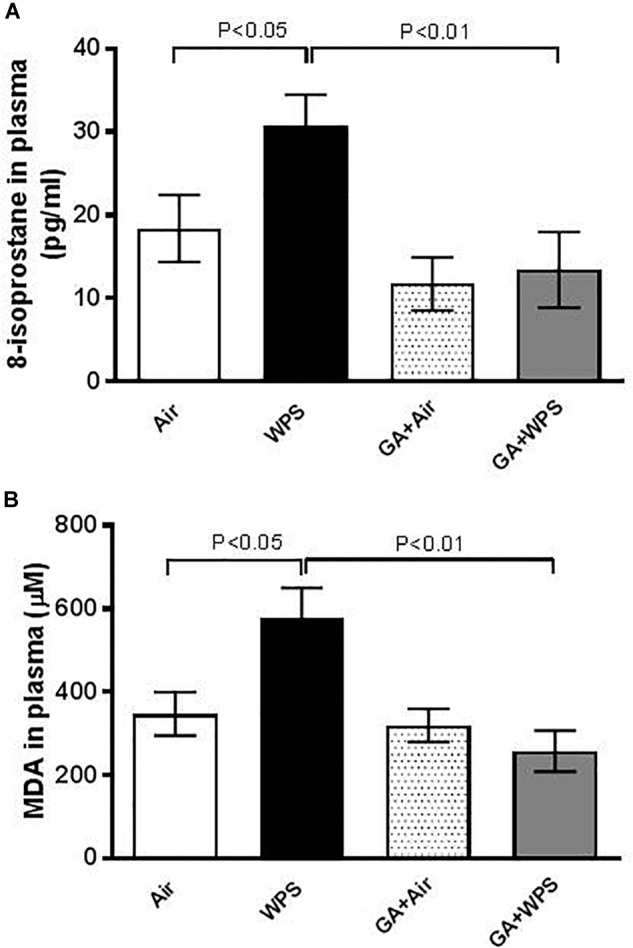
Plasma concentrations of 8-isoprostane **(A)** at the end of 1 month exposure period to either air (*n* = 8) or WPS (*n* = 7) or GA administration (15% *w*/*v* in the drinking water) + air (*n* = 8) or GA + WPS (*n* = 8). Malondialdehyde (MDA, **B**) concentrations in the plasma at the end of 1 month exposure period to either air (*n* = 5) or WPS (*n* = 6) or GA administration (15% *w*/*v* in the drinking water) + air (*n* = 6) or GA + WPS (*n* = 7). Data are mean ± SEM.

Compared with the air-exposed group, the plasma concentrations of markers of lipid peroxidation, 8-isoprostane and MDA, were significantly elevated following exposure to WPS (*P* < 0.05). GA caused a significant (*P* < 0.01) abrogation of the increase of plasma concentration of 8-isoprostane and MDA induced by WPS (Figure 5).

### Effect of GA on WPS-Induced Inflammation in Heart Homogenates

**FIGURE 6:**
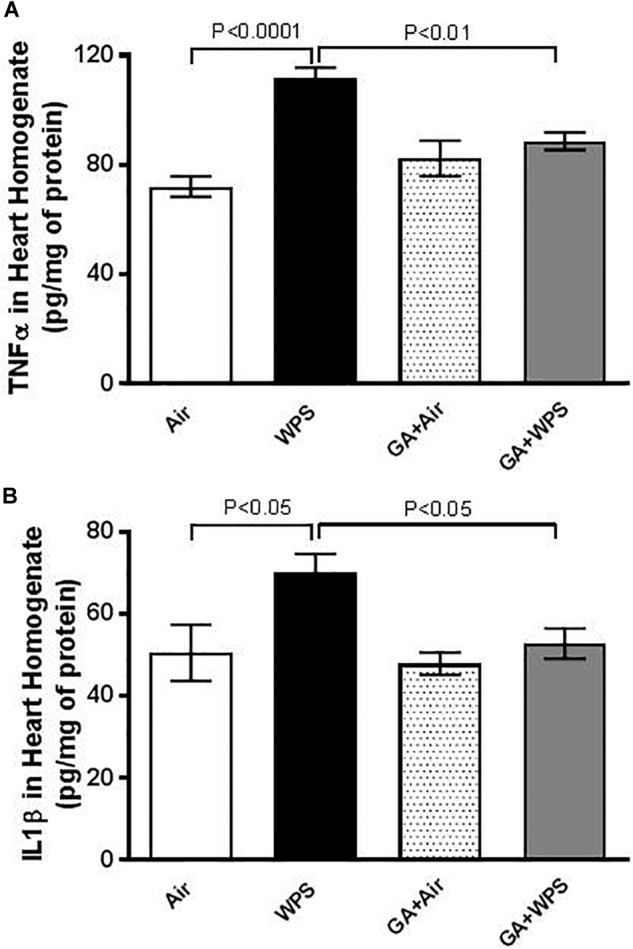
Heart homogenate concentrations of tumor necrosis factor α (TNFα, **A**) at the end of 1 month exposure period to either air (*n* = 8) or WPS (*n* = 8) or GA administration (15% *w*/*v* in the drinking water) + air (*n* = 6) or GA + WPS (*n* = 6). Interleukin-1β (IL-1 β, **B**) concentrations in heart homogenates at the end of 1 month exposure period to either air (*n* = 8) or WPS (*n* = 7) or GA administration (15% *w*/*v* in the drinking water) + air (*n* = 7) or GA + WPS (*n* = 8). Data are mean ± SEM.

Figure 6 shows that exposure to WPS caused an augmentation in the concentrations of TNFα (*P* < 0.0001) and IL-1β (*P* < 0.05) in heart homogenates. Compared with the WPS group, mice treated with GA and exposed to WPS showed a potent reduction in the increase in TNFα (*P* < 0.01) and IL-1β (*P* < 0.05) concentrations in heart homogenates.

### Effect of GA on WPS-Induced Oxidative Stress in Heart Homogenates

**FIGURE 7:**
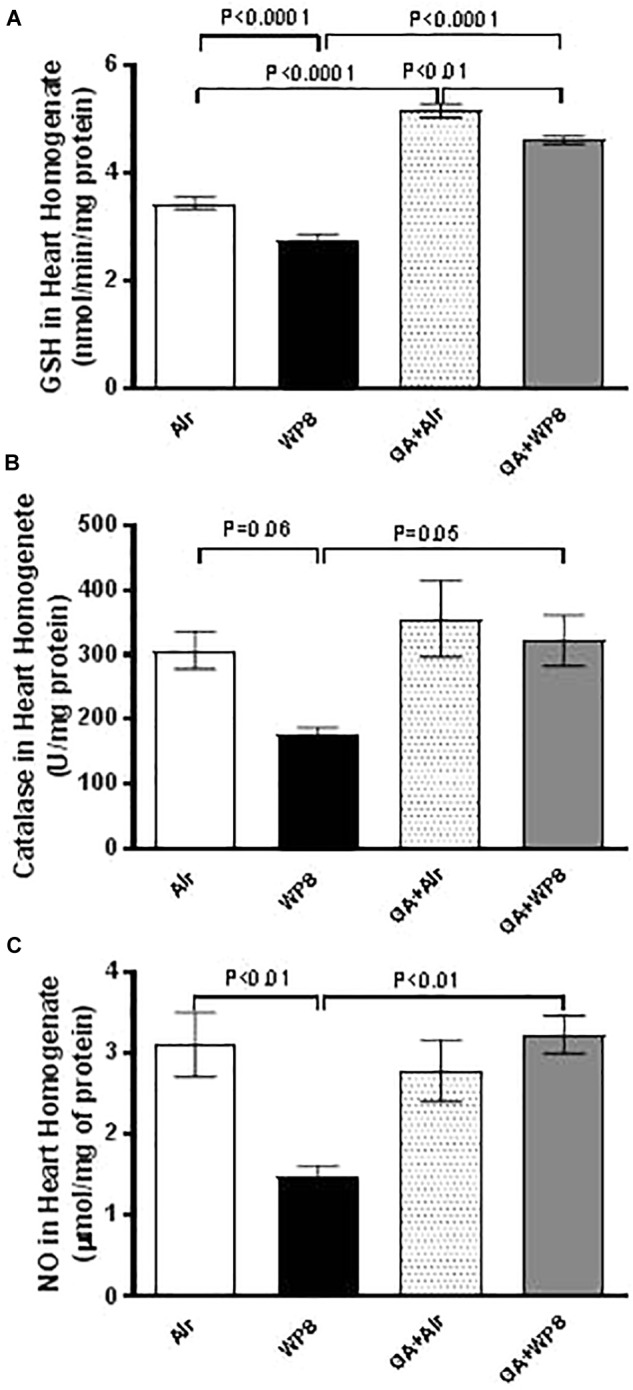
Heart homogenate concentrations of reduced glutathione (GSH, **A**) at the end of 1 month exposure period to either air (*n* = 8) or WPS (*n* = 8) or GA administration (15% *w*/*v* in the drinking water) + air (*n* = 8) or GA + WPS (*n* = 8). Catalase activity **(B)** in heart homogenates at the end of 1 month exposure period to either air (*n* = 5) or WPS (*n* = 5) or GA administration (15% *w*/*v* in the drinking water) + air (*n* = 5) or GA + WPS (*n* = 5). Total nitric oxide activity (NO, **C**) in heart homogenates at the end of 1 month exposure period to either air (*n* = 8) or WPS (*n* = 6) or GA administration (15% *w*/*v* in the drinking water) + air (*n* = 8) or GA + WPS (*n* = 8). Data are mean ± SEM.

As shown in Figure 7A,C compared with the air-exposed group, GSH (0.0001) and total NO (0.01) levels in heart homogenates were significantly decreased by WPS exposure. Figure 7B shows that catalase activity was also decreased in mice exposed to WPS compared with those exposed to air, however, this decrease failed to reach statistical significance (*P* = 0.06). GA administration restored the decrease in the GSH, catalase and NO levels induced by WPS exposure.

### Effect of WPS on Heart Histology and Expression of Nrf2, and Influence of GA Treatment

**FIGURE 8:**
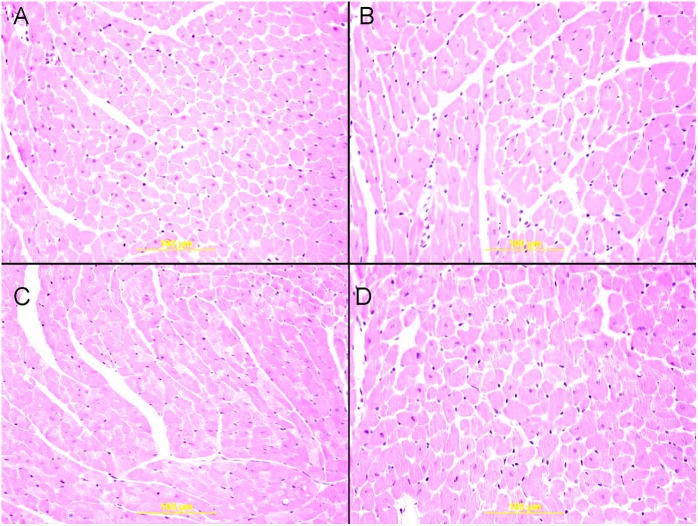
Representative light microscopy sections of H and E stained heart tissues of mice at the end of 1 month exposure period to either air or WPS with or without GA administration (15% *w*/*v* in the drinking water). **(A)** Representative heart sections of the left ventricle obtained from air-exposed mice. **(B)** Representative heart sections of the left ventricle obtained from air-exposed mice treated with GA. **(C)** Representative heart sections of the left ventricle obtained from WPS-exposed mice. **(D)** Representative heart sections of the left ventricle obtained from WPS-exposed mice treated with GA.

There were no significant morphological changes in H&E stained sections between air, GA+air, WPS and GA+WPS groups (Figure 8).

**FIGURE 9:**
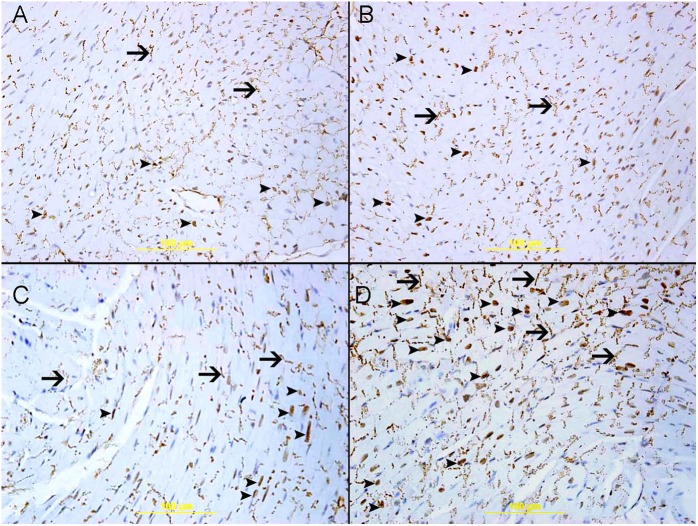
Immunohistochemical analysis of the heart tissue sections for the detection of nuclear factor erythroid-derived 2-like 2 (Nrf2) mice at the end of 1 month exposure period to either air or WPS with or without GA administration (15% *w*/*v* in the drinking water). **(A)** Representative section of the heart of air-exposed mice showing focal mild nuclear expression of Nrf2 by cardiac myocytes (arrow head). There is also cytoplasmic expression of Nrf2 (arrow). **(B)** Representative section of the left ventricle of GA+air group showing nuclear expression of Nrf2 by cardiac myocytes (arrow head). There is also cytoplasmic expression of Nrf2 (arrow). **(C)** Representative section of the left ventricle of WPS-exposed mice showing nuclear expression of Nrf2 by cardiac myocytes (arrow head). There is also cytoplasmic expression of NRF2 (arrow). **(D)** Representative section of the left ventricle of GA+WPS group showing potentiation in nuclear expression of Nrf2 by cardiac myocytes (arrow head). There is also cytoplasmic expression of Nrf2 (arrow). Immunoperoxidase streptavidin biotin method.

Figure 9 illustrates that there is nuclear and cytoplasmic expression of Nrf2 by cardiac myocytes and endothelial cells in the heart sections of air, WPS, GA+air and GA+WPS (Figure 9), with various intensity and distribution. The nuclear expression of Nrf2 correlates with its antioxidant transcriptional action whereas its cytoplasmic expression does not count for its transcriptional activity.

The air-exposed group showed mild nuclear expression of Nrf2 by a few cardiomyocytes and endothelial cells and scored 1 (Figure 9A). Likewise, there was cytoplasmic expression of Nrf2 by cardiac myocytes. Compared with the air exposed group, the GA+air group showed an elevation in the nuclear expression of Nrf2 by cardiomyocytes and endothelial cells and scored 2 (Figure 9B). The WPS exposed group displayed an augmentation in the nuclear expression of Nrf2 by cardiomyocytes and endothelial cells when compared with the air-exposed group and scored 2 (Figure 9C). The group of mice treated with GA and exposed to WPS exhibited an elevation in the nuclear expression of Nrf2 by cardiomyocyte and endothelial cells when compared with mice exposed either to WPS or those exposed to air and scored 3 (Figure 9D).

### Effect of GA on WPS-Induced Increase in DNA Damage and Cleaved Caspase 3 in Heart Homogenates

**FIGURE 10:**
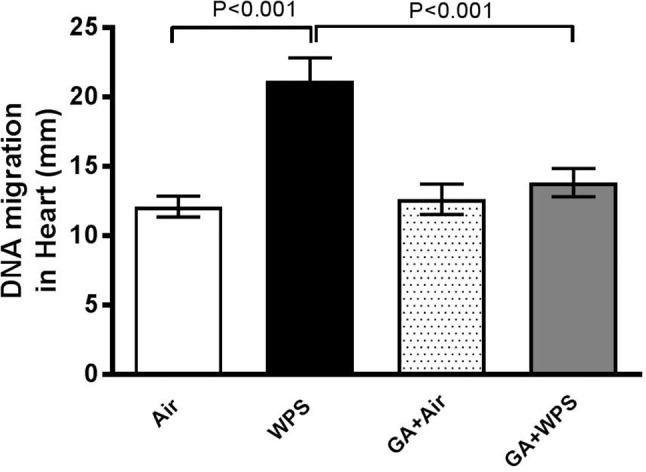
DNA migration (mm) in the heart tissue of mice assessed by Comet assay at the end of 1 month exposure period to either air (*n* = 5) or WPS (*n* = 5) or GA administration (15% *w*/*v* in the drinking water) + air (*n* = 5) or GA + WPS (*n* = 5). Data are mean ± SEM.

Figure 10 shows the impact of WPS exposure on heart DNA damage and the effect of GA treatment thereon. Compared with the air-exposed group, WPS exposure induced a significant augmentation in DNA migration (*P* < 0.001). The latter effect was significantly reduced by GA administration (*P* < 0.001).

**FIGURE 11:**
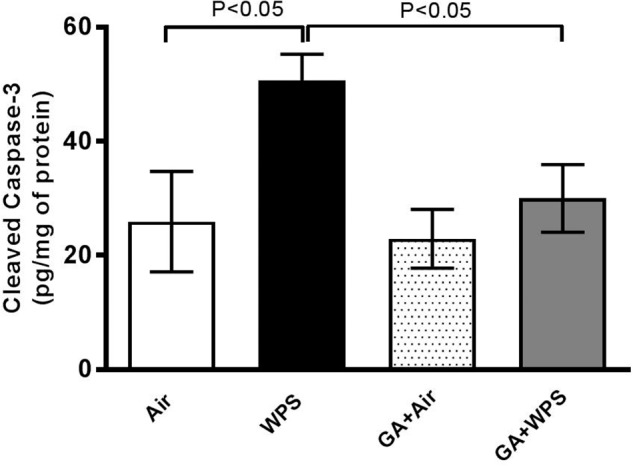
Heart homogenate concentrations of cleaved caspase-3 at the end of 1 month exposure period to either air (*n* = 6) or WPS (*n* = 8) or GA administration (15% *w*/*v* in the drinking water) + air (*n* = 6) or GA + WPS (*n* = 7). Data are mean ± SEM.

Likewise, compared with the air-exposed group, WPS exposure caused a significant increase in the concentration of cleaved caspase-3 (*P* < 0.05). Treatment of mice with GA induced a significant reduction in the augmentation of the concentration of cleaved caspase-3 induced by WPS exposure (*P* < 0.05) (Figure 11).

## Discussion

Here, we showed that GA alleviated WPS-induced thrombotic disorders, systemic oxidative stress and increase in SBP. Moreover, we found that cardiac inflammation, oxidative stress, DNA damage and apoptosis triggered by WPS were significantly mitigated by GA treatment by a mechanism which involves activation of the Nrf2 signaling pathway.

Before the nineties, WPS was not popular, was mainly confined to the Middle Eastern societies, and was mainly practiced by older men. However, after that, the epidemiology of WPS began to change causing its spread among the youth and to other countries including Europe and United States (Maziak et al., 2015).

The efficacy of the use of the prebiotic GA has been established by various clinical and experimental studies (Gamal el-din et al., 2003; Babiker et al., 2012; Ali et al., 2013b, 2014; Elamin et al., 2017; Kaddam et al., 2017; Al Za’abi et al., 2018). While the exact mechanism by which GA exerts its protective effect is not fully understood, it has been suggested that its beneficial effects are mediated through its antioxidant and anti-inflammatory actions (Ali et al., 2013a).

For the first time, the present study was designed to assess the possible protective effect of GA on the cardiovascular toxicity induced by WPS. Our data show that GA administration induced a significant ameliorative effect on WPS exposure-induced impairment of coagulation. In fact, GA mitigated the prothrombotic effects in pial arterioles and venules *in vivo*, platelet aggregation in whole blood *in vitro* and shortening of PT induced by WPS exposure. Fibrinogen is an acute-phase protein which has been reported to increase blood viscosity and induce thrombotic complications (Su et al., 2006). It is well known that PAI-1 is an efficient inhibitor of fibrinolysis and its augmentation has been linked to a variety of clinical conditions including obesity, insulin resistance, psychosocial stress, reduced immune responses, increased inflammation and thrombotic events (Cesari et al., 2010). Moreover, it has been shown that plasma PAI-1 and fibrinogen levels were significantly higher among smokers (Barua and Ambrose, 2013; Delgado et al., 2015). Remarkably, we have also found here that GA has prevented WPS-induced augmentation of PAI-1 and fibrinogen. This novel effect substantiates the ameliorative effect of GA against WPS induced thrombotic events *in vivo* and *in vitro*. Clinical and experimental studies have reported that exposure to WPS causes a significant increase in SBP (Hakim et al., 2011; Nemmar et al., 2013b, 2017b; Kadhum et al., 2014). Our data showed that exposure to WPS induced a significant increase in SBP, and that the treatment with GA significantly reduced this effect. It has been hypothesized that oxidative stress is a key player in the pathogenesis of thrombosis and hypertension (Baradaran et al., 2014; Wilson et al., 2017). To gain more insight into the mechanisms responsible for this increase of thrombosis and SBP, we measured markers of oxidative stress, namely MDA and 8-isoprostane, which are well-known markers of lipid peroxidation. Our data show that the concentrations of MDA and 8-isoprostane were increased by WPS, and that GA administration averted this increase. It has been shown that exposure to particulate air pollution induces oxidative stress which in turn triggers prothrombotic events, and that treatment with antioxidant alleviated this effect (Nemmar et al., 2009, 2011). Moreover, our finding corroborates the studies which have reported that GA is effective in reducing the increase in SBP in patients with type-2 diabetes and in rats with adenine-induced chronic renal failure (Ali et al., 2011; Babiker et al., 2018).

To assess further the mechanism underlying the pathophysiological impact of WPS on the heart and the possible protective effect of GA thereon, we have measured markers of inflammation including TNFα and IL-1β and antioxidants comprising GSH, catalase and NO in heart homogenates. Our data show that GA supplementation significantly prevented the increase of the pro-inflammatory cytokine TNFα and IL-1β caused by WPS exposure. Moreover, GA restored the decrease in the GSH, catalase and NO. The decrease of these biomarkers indicates their depletion in the course of the breakdown of free radicals, and their return back to the control levels after the treatment with GA is suggestive that GA is a potent natural anti-inflammatory and antioxidant agent. These findings corroborate recent clinical and experimental studies which have reported that GA exerts antioxidant effects in patients with sickle cell anemia, and both anti-inflammatory and antioxidant actions in rats with chronic renal failure (Ali et al., 2013a; Kaddam et al., 2017; Al Za’abi et al., 2018).

Our data did not show any effect of WPS on heart morphology. This finding obtained at the 1 month-time point is consistent with our recent study which showed the absence of morphological changes in the heart of mice exposed for 6 month to WPS (Nemmar et al., 2017b). Nrf2 is a critical transcription factor which plays a major role in activating antioxidant enzymes to respond to oxidative stress (Barancik et al., 2016). When the antioxidants are activated to remove reactive oxygen species, the Nrf2 is released from the regulatory Keap1–Nrf2 complex and translocates from the cytoplasm to the nucleus, where it binds to the antioxidant response element, a regulatory enhancer region within gene promoters. This binding triggers the production of antioxidant enzymes which protect the cells from oxidative stress (Barancik et al., 2016). The effect of WPS on Nrf2 expression in heart has not been investigated so far. Similarly to the GSH concentrations which were elevated in the hearts of mice treated with GA and exposed to air compared with those exposed to air only, the nuclear expression of Nrf2 by cardiomyocyte and endothelial cells was also increased in GA+air group compared with air group. The latter findings suggest that GA administration has the ability to increase the levels of antioxidants even in healthy mice exposed to air.

Moreover, we showed that WPS induced an increase in the nuclear expression of Nrf2 by cardiomyocytes and endothelial cells when compared with the air-exposed group. Interestingly, in mice treated with GA and exposed to WPS, we found a substantial increase in the nuclear expression of Nrf2 by cardiomyocyte and endothelial cells when compared with either WPS or air-exposed groups. The overexpression of Nrf2 in GA+WPS along with the increase of levels of GSH, catalase and total NO and decrease in the concentrations of pro-inflammatory cytokines TNFα and IL-1β suggests that the protective effect of GA against WPS-induced cardiovascular toxicity involves Nrf2 activation. Nrf2 has been shown to be an important therapeutic target in several pathophysiological situations for its activation of anti-oxidant enzymes (Barancik et al., 2016). Also, several Nrf2 activators displayed potent anti-inflammatory characteristics (Barancik et al., 2016). It has been recently reported that the anti-oxidant, anti-inflammatory and anti-fibrotic effects of chrysophanol, an anthraquinone compound extracted from plants of the *Rheum* genus, against high fat-induced cardiac injury are regulated by Nrf2 expression (Lian et al., 2017). Additionally, it has been shown that nootkatone, a constituent of grapefruit with antioxidant and anti-inflammatory properties, exerts its protective effect against diesel exhaust particles-induced cardiovascular toxicity through a mechanism involving Nrf2 activation (Nemmar et al., 2018a). With respect to pulmonary injury induced by WPS, it has been recently demonstrated that exercise training possesses an ameliorative effect involving activating the Nrf2 signaling pathway (Nemmar et al., 2018b).

It is well established that oxidative stress may cause DNA oxidation injury (Savitskaya and Onishchenko, 2015). Moreover, it is also well-known that reactive oxygen species can trigger apoptosis characteristically via caspase-3 activation (Savitskaya and Onishchenko, 2015). Our data show that WPS exposure elicited DNA damage, and that this effect has been potently prevented by GA administration. In line with the latter finding, we have also demonstrated that WPS exposure caused a significant increase in cleaved caspase-3, indicating the occurrence of apoptosis and that this effect has been completely averted in the GA+WPS group. Our data are in agreement with a previous study performed in a rat model of renal failure which showed that GA inhibited DNA damage and apoptosis in the kidneys (Ali et al., 2013a).

In conclusion, our experimental data obtained in mice show, for the first time, that GA treatment prevented WPS-induced thrombotic events *in vivo* and *in vitro*, increase in SBP, systemic and cardiac oxidative stress and inflammation, DNA damage and apoptosis through a mechanism involving Nrf2 activation. Our study provides evidence that the use of GA has the potential to mitigate potently the cardiovascular pathophysiological effects caused by WPS. Additional studies are required to assess the possible effect of burning of the charcoal disk and the additives that are supplemented to tobacco added in shisha such as flavorings, and to verify whether and to what extent our findings could be extrapolated to other experimental models and human patients.

## Author Contributions

All authors have read and approved the manuscript. AN designed, planned, supervised the experiments and wrote the article. SA-S performed the histology part of the work. SB and PY performed the experiments. BA contributed in the design of the study and the writing of the manuscript.

## Conflict of Interest Statement

The authors declare that the research was conducted in the absence of any commercial or financial relationships that could be construed as a potential conflict of interest.

## References

[B1] Aickin M. Gensler H. (1996). Adjusting for multiple testing when reporting research results: the Bonferroni vs Holm methods. *Am. J. Public Health* 86 726–728. 10.2105/AJPH.86.5.726 8629727 PMC1380484

[B2] Al Ghobain M. Ahmed A. Abdrabalnabi Z. Mutairi W. Al K. A. (2018). Prevalence of and attitudes to waterpipe smoking among Saudi Arabian physicians. *East Mediterr. Health J.* 24 277–282. 10.26719/2018.24.3.277 29908023

[B3] Al Za’abi M. Al Salam S. Al Suleimani Y. Manoj P. Nemmar A. Ali B. H. (2018). Gum acacia improves renal function and ameliorates systemic inflammation, oxidative and nitrosative stress in streptozotocin-induced diabetes in rats with adenine-induced chronic kidney disease. *Cell. Physiol. Biochem.* 45 2293–2304. 10.1159/000488176 29550811

[B4] Ali B. H. Al Balushi K. A. Ashique M. Shalaby A. Al Kindi M. A. Adham S. A. (2017). Chronic water-pipe smoke exposure induces injurious effects to reproductive system in male mice. *Front. Physiol.* 8:158. 10.3389/fphys.2017.00158 28420996 PMC5378788

[B5] Ali B. H. Al-Husseni I. Beegam S. Al-Shukaili A. Nemmar A. Schierling S. (2013a). Effect of gum arabic on oxidative stress and inflammation in adenine-induced chronic renal failure in rats. *PLoS One* 8:e55242. 10.1371/journal.pone.0055242 23383316 PMC3562323

[B6] Ali B. H. Al-Salam S. Al Za’abi M. Waly M. I. Ramkumar A. Beegam S. (2013b). New model for adenine-induced chronic renal failure in mice, and the effect of gum acacia treatment thereon: comparison with rats. *J. Pharmacol. Toxicol. Methods* 68 384–393. 10.1016/j.vascn.2013.05.001 23669035

[B7] Ali B. H. Inuwa I. Al Za’abi M. Al Bahlani S. Al Issaei H. Ramkumar A. (2014). Renal and myocardial histopathology and morphometry in rats with adenine - induced chronic renal failure: influence of gum acacia. *Cell. Physiol. Biochem.* 34 818–828. 10.1159/000363045 25171124

[B8] Ali B. H. Ziada A. Al H. I Beegam S. Al-Ruqaishi B. Nemmar A. (2011). Effect of Acacia gum on blood pressure in rats with adenine-induced chronic renal failure. *Phytomedicine* 18 1176–1180. 10.1016/j.phymed.2011.03.005 21741228

[B9] Ali B. H. Ziada A. Blunden G. (2009). Biological effects of gum arabic: a review of some recent research. *Food Chem. Toxicol.* 47 1–8. 10.1016/j.fct.2008.07.001 18672018

[B10] Babiker R. Elmusharaf K. Keogh M. B. Saeed A. M. (2018). Effect of gum arabic (Acacia Senegal) supplementation on visceral adiposity index (VAI) and blood pressure in patients with type 2 diabetes mellitus as indicators of cardiovascular disease (CVD): a randomized and placebo-controlled clinical trial. *Lipids Health Dis.* 17:56. 10.1186/s12944-018-0711-y 29558953 PMC5859434

[B11] Babiker R. Merghani T. H. Elmusharaf K. Badi R. M. Lang F. Saeed A. M. (2012). Effects of gum arabic ingestion on body mass index and body fat percentage in healthy adult females: two-arm randomized, placebo controlled, double-blind trial. *Nutr. J.* 11:111. 10.1186/1475-2891-11-111 23241359 PMC3570285

[B12] Baradaran A. Nasri H. Rafieian-Kopaei M. (2014). Oxidative stress and hypertension: possibility of hypertension therapy with antioxidants. *J. Res. Med. Sci.* 19 358–367. 25097610 PMC4115353

[B13] Barancik M. Gresova L. Bartekova M. Dovinova I. (2016). Nrf2 as a key player of redox regulation in cardiovascular diseases. *Physiol. Res.* 65(Suppl. 1), S1–S10. 27643930 10.33549/physiolres.933403

[B14] Barua R. S. Ambrose J. A. (2013). Mechanisms of coronary thrombosis in cigarette smoke exposure. *Arterioscler. Thromb. Vasc. Biol.* 33 1460–1467. 10.1161/ATVBAHA.112.300154 23685556

[B15] Cesari M. Pahor M. Incalzi R. A. (2010). Plasminogen activator inhibitor-1 (PAI-1): a key factor linking fibrinolysis and age-related subclinical and clinical conditions. *Cardiovasc. Ther.* 28:e0072–91. 10.1111/j.1755-5922.2010.00171.x 20626406 PMC2958211

[B16] de Souza M. F. Goncales T. A. Steinmetz A. Moura D. J. Saffi J. Gomez R. (2014). Cocaine induces DNA damage in distinct brain areas of female rats under different hormonal conditions. *Clin. Exp. Pharmacol. Physiol.* 41 265–269. 10.1111/1440-1681.12218 24552452

[B17] Delgado G. E. Siekmeier R. Kramer B. K. Marz W. Kleber M. E. (2015). Plasma fibrinolysis parameters in smokers and non-smokers of the ludwigshafen risk and cardiovascular health (LURIC) study. *Adv. Exp. Med. Biol.* 858 69–77. 10.1007/5584_2015_127 25786403

[B18] El Sabban F. Radwan G. M. (1997). Influence of garlic compared to aspirin on induced photothrombosis in mouse pial microvessels, in vivo. *Thromb. Res.* 88 193–203. 10.1016/S0049-3848(97)00230-2 9361372

[B19] Elamin S. Alkhawaja M. J. Bukhamsin A. Y. Idris M. A. S. Abdelrahman M. M. Abutaleb N. K. (2017). Gum arabic reduces C-reactive protein in chronic kidney disease patients without affecting urea or indoxyl sulfate levels. *Int. J. Nephrol.* 2017:9501470. 10.1155/2017/9501470 28589039 PMC5446885

[B20] Gamal el-din A. M. Mostafa A. M. Al-Shabanah O. A. Al-Bekairi A. M. Nagi M. N. (2003). Protective effect of arabic gum against acetaminophen-induced hepatotoxicity in mice. *Pharmacol. Res.* 48 631–635. 10.1016/S1043-6618(03)00226-3 14527829

[B21] Grant A. Morrison R. Dockrell M. J. (2014). Prevalence of waterpipe (Shisha, Narghille, Hookah) use among adults in great britain and factors associated with waterpipe use: data from cross-sectional online surveys in 2012 and 2013. *Nicotine Tob. Res.* 16 931–938. 10.1093/ntr/ntu015 24550183

[B22] Hakim F. Hellou E. Goldbart A. Katz R. Bentur Y. Bentur L. (2011). The acute effects of water-pipe smoking on the cardiorespiratory system. *Chest* 139 775–781. 10.1378/chest.10-1833 21030492

[B23] Hartmann A. Speit G. (1997). The contribution of cytotoxicity to DNA-effects in the single cell gel test (comet assay). *Toxicol. Lett.* 90 183–188. 10.1016/S0378-4274(96)03847-7 9067486

[B24] Holm S. (1979). A simple sequentially rejective multiple test procedure. *Scand. J. Statist.* 6 65–70.

[B25] Hooper L. Kroon P. A. Rimm E. B. Cohn J. S. Harvey I. Le Cornu K. A. (2008). Flavonoids, flavonoid-rich foods, and cardiovascular risk: a meta-analysis of randomized controlled trials. *Am. J. Clin. Nutr.* 88 38–50. 10.1093/ajcn/88.1.38 18614722

[B26] Islam M. A. Alam F. Solayman M. Khalil M. I. Kamal M. A. Gan S. H. (2016). Dietary phytochemicals: natural swords combating inflammation and oxidation-mediated degenerative diseases. *Oxid. Med. Cell. Longev.* 2016:5137431. 10.1155/2016/5137431 27721914 PMC5046019

[B27] Jensen P. D. Cortes R. Engholm G. Kremers S. Gislum M. (2010). Waterpipe use predicts progression to regular cigarette smoking among Danish youth. *Subst. Use Misuse* 45 1245–1261. 10.3109/10826081003682909 20441461

[B28] Kaddam L. Fadl-Elmula I. Eisawi O. A. Abdelrazig H. A. Salih M. A. Lang F. (2017). Gum Arabic as novel anti-oxidant agent in sickle cell anemia, phase II trial. *BMC Hematol.* 17:4. 10.1186/s12878-017-0075-y 28331623 PMC5356407

[B29] Kadhum M. Jaffery A. Haq A. Bacon J. Madden B. (2014). Measuring the acute cardiovascular effects of shisha smoking: a cross-sectional study. *JRSM Open* 5:2054270414531127. 10.1177/2054270414531127 25057403 PMC4100228

[B30] Lian Y. Xia X. Zhao H. Zhu Y. (2017). The potential of chrysophanol in protecting against high fat-induced cardiac injury through Nrf2-regulated anti-inflammation, anti-oxidant and anti-fibrosis in Nrf2 knockout mice. *Biomed. Pharmacother.* 93 1175–1189. 10.1016/j.biopha.2017.05.148 28738526

[B31] Maziak W. Ben T. Z. Jawad M. Afifi R. Nakkash R. Akl E. A. (2017). Consensus statement on assessment of waterpipe smoking in epidemiological studies. *Tob. Control* 26 338–343. 10.1136/tobaccocontrol-2016-052958 27165995 PMC5104675

[B32] Maziak W. Taleb Z. B. Bahelah R. Islam F. Jaber R. Auf R. (2015). The global epidemiology of waterpipe smoking. *Tob. Control* 24(Suppl. 1), i3–i12. 10.1136/tobaccocontrol-2014-051903 25298368 PMC4345835

[B33] Nemmar A. Al Hemeiri A. Al Hammadi N. Yuvaraju P. Beegam S. Yasin J. (2015a). Early pulmonary events of nose-only water pipe (shisha) smoking exposure in mice. *Physiol. Rep.* 3:e12258. 10.14814/phy2.12258 25780090 PMC4393146

[B34] Nemmar A. Yuvaraju P. Beegam S. Ali B. H. (2015b). Short-term nose-only water-pipe (shisha) smoking exposure accelerates coagulation and causes cardiac inflammation and oxidative stress in mice. *Cell. Physiol. Biochem.* 35 829–840. 10.1159/000369741 25634761

[B35] Nemmar A. Al Salam S. Dhanasekaran S. Sudhadevi M. Ali B. H. (2009). Pulmonary exposure to diesel exhaust particles promotes cerebral microvessel thrombosis: protective effect of a cysteine prodrug l-2-oxothiazolidine-4-carboxylic acid. *Toxicology* 263 84–92. 10.1016/j.tox.2009.06.017 19560508

[B36] Nemmar A. Albarwani S. Beegam S. Yuvaraju P. Yasin J. Attoub S. (2014). Amorphous silica nanoparticles impair vascular homeostasis and induce systemic inflammation. *Int. J. Nanomedicine* 9 2779–2789. 10.2147/IJN.S52818 24936130 PMC4047982

[B37] Nemmar A. Al-Salam S. Beegam S. Yuvaraju P. Ali B. H. (2018a). Thrombosis, systemic and cardiac oxidative stress and DNA damage induced by pulmonary exposure to diesel exhaust particles, and the effect of nootkatone thereon. *Am. J. Physiol. Heart Circ. Physiol.* 314 H917–H927. 10.1152/ajpheart.00313.2017 29351455

[B38] Nemmar A. Al-Salam S. Yuvaraju P. Beegam S. Ali B. H. (2018b). Exercise training mitigates water pipe smoke exposure-induced pulmonary impairment via inhibiting NF-kappaB and activating Nrf2 signalling pathways. *Oxid. Med. Cell. Longev.* 2018:7459612. 10.1155/2018/7459612 29692875 PMC5859847

[B39] Nemmar A. Al-Salam S. Beegam S. Yuvaraju P. Oulhaj A. Ali B. H. (2017a). Water-pipe smoke exposure-induced circulatory disturbances in mice, and the influence of betaine supplementation thereon. *Cell. Physiol. Biochem.* 41 1098–1112. 10.1159/000464117 28245471

[B40] Nemmar A. Al-Salam S. Yuvaraju P. Beegam S. Yasin J. Ali B. H. (2017b). Chronic exposure to water-pipe smoke induces cardiovascular dysfunction in mice. *Am. J. Physiol. Heart Circ. Physiol.* 312 H329–H339. 10.1152/ajpheart.00450.2016 27940964

[B41] Nemmar A. Al-Salam S. Yuvaraju P. Beegam S. Yasin J. Ali B. H. (2016a). Chronic exposure to water-pipe smoke induces alveolar enlargement, DNA damage and impairment of lung function. *Cell. Physiol. Biochem.* 38 982–992. 10.1159/000443050 26938718

[B42] Nemmar A. Yuvaraju P. Beegam S. Yasin J. Kazzam E. E. Ali B. H. (2016b). Oxidative stress, inflammation, and DNA damage in multiple organs of mice acutely exposed to amorphous silica nanoparticles. *Int. J. Nanomedicine* 11 919–928. 10.2147/IJN.S92278 27022259 PMC4788369

[B43] Nemmar A. Al-Salam S. Zia S. Marzouqi F. Al-Dhaheri A. Subramaniyan D. (2011). Contrasting actions of diesel exhaust particles on the pulmonary and cardiovascular systems and the effects of thymoquinone. *Br. J. Pharmacol.* 164 1871–1882. 10.1111/j.1476-5381.2011.01442.x 21501145 PMC3246712

[B44] Nemmar A. Melghit K. Ali B. H. (2008). The acute proinflammatory and prothrombotic effects of pulmonary exposure to rutile TiO2 nanorods in rats. *Exp. Biol. Med.* 233 610–619. 10.3181/0706-RM-165 18375825

[B45] Nemmar A. Raza H. Subramaniyan D. Yasin J. John A. Ali B. H. (2013a). Short-term systemic effects of nose-only cigarette smoke exposure in mice: role of oxidative stress. *Cell. Physiol. Biochem.* 31 15–24. 10.1159/000343345 23343613

[B46] Nemmar A. Yuvaraju P. Beegam S. John A. Raza H. Ali B. H. (2013b). Cardiovascular effects of nose-only water-pipe smoking exposure in mice. *Am. J. Physiol. Heart Circ. Physiol.* 305 H740–H746. 10.1152/ajpheart.00200.2013 23812392

[B47] Olive P. L. Banath J. P. Fjell C. D. (1994). DNA strand breakage and DNA structure influence staining with propidium iodide using the alkaline comet assay. *Cytometry* 16 305–312. 10.1002/cyto.990160404 7527314

[B48] R Core Team (2014). *R: A language and environment for statistical Computing (Version 3.0.3)*. Vienna, R Foundation for Statistical Computing Available at: http://www.R-project.org/

[B49] Salloum R. G. Thrasher J. F. Kates F. R. Maziak W. (2015). Water pipe tobacco smoking in the united states: findings from the national adult tobacco survey. *Prev. Med.* 71 88–93. 10.1016/j.ypmed.2014.12.012 25535678 PMC4423406

[B50] Savitskaya M. A. Onishchenko G. E. (2015). Mechanisms of apoptosis. *Biochemistry* 80 1393–1405. 26615431 10.1134/S0006297915110012

[B51] Sibai A. M. Tohme R. A. Almedawar M. M. Itani T. Yassine S. I. Nohra E. A. (2014). Lifetime cumulative exposure to waterpipe smoking is associated with coronary artery disease. *Atherosclerosis* 234 454–460. 10.1016/j.atherosclerosis.2014.03.036 24814409

[B52] Su T. C. Chan C. C. Liau C. S. Lin L. Y. Kao H. L. Chuang K. J. (2006). Urban air pollution increases plasma fibrinogen and plasminogen activator inhibitor-1 levels in susceptible patients. *Eur. J. Cardiovasc. Prev. Rehabil.* 13 849–852. 10.1097/01.hjr.0000219116.25415.c4 17001229

[B53] Tsikas D. (2005). Methods of quantitative analysis of the nitric oxide metabolites nitrite and nitrate in human biological fluids. *Free Radic. Res.* 39 797–815. 10.1080/10715760500053651 16036360

[B54] Wennmalm A. Benthin G. Edlund A. Jungersten L. Kieler-Jensen N. Lundin S. (1993). Metabolism and excretion of nitric oxide in humans. An experimental and clinical study. *Circ. Res.* 73 1121–1127. 10.1161/01.RES.73.6.1121 8222083

[B55] Wilson S. J. Miller M. R. Newby D. E. (2017). Effects of diesel exhaust on cardiovascular function and oxidative stress. *Antioxid. Redox. Signal.* 28 819–836. 10.1089/ars.2017.7174 28540736

